# A Review of Medical Data Sharing Initiatives With a Focus on the Use of Blockchain Technologies: Qualitative Comparative Analysis

**DOI:** 10.2196/63575

**Published:** 2026-04-22

**Authors:** James Cunningham, Nigel Davies, Sarah Devaney, Mike Harding, Søren Holm, Victoria Neumann, Clare Smith, John Ainsworth

**Affiliations:** 1 School of Health Sciences, University of Manchester, Oxford Road, Manchester, M13 9PL, United Kingdom, 44 0161 306 6000; 2 School of Computing and Communications, Lancaster University, Lancaster, United Kingdom; 3 Centre for Social Ethics and Policy, University of Manchester, Manchester, United Kingdom; 4 Civic Health Innovation Labs, University of Liverpool, Liverpool, United Kingdom

**Keywords:** blockchain, medical data sharing, data governance, sustainability models, privacy, security, transparency, data provision, ethics

## Abstract

**Background:**

Medical data sharing initiatives are crucial for advancing research, improving patient outcomes, and fostering innovation in health care. With the advent of blockchain technology, there has been significant interest in exploring its potential to enhance the security, transparency, and efficiency of medical data sharing.

**Objective:**

This study aimed to examine a selected set of blockchain-based medical data sharing initiatives, focusing on their governance, incentive structures, ownership models, business approaches, transaction mechanisms, and sustainability strategies. The analysis explored patterns in operational status and longevity, providing insight into the factors shaping these initiatives. The objective was to identify common characteristics and contextual factors that may influence their development and persistence.

**Methods:**

The study used snowball sampling to identify a selection of primarily blockchain-based medical data sharing initiatives, drawing from academic literature, web searches, and expert consultations. To examine structural and operational patterns, initiatives were selected based on the availability of sufficient public documentation for systematic classification. Each initiative was categorized by governance, incentives, ownership, business models, transaction mechanisms, and sustainability strategies. A follow-up assessment examined operational status over time. The analysis applied qualitative comparative analysis to identify common structural features and relationships between governance, incentives, and sustainability.

**Results:**

The survey identified 42 initiatives, categorizing them based on ownership, governance, business, incentive, transaction, and sustainability models. These categories were systematically identified and assigned numerical values to facilitate fuzzy-set qualitative comparative analysis. The base model, run at an inclusion threshold of 0.65, identified multiple configurations associated with sustained initiative activity, highlighting the role of governance mechanisms and transaction structures in supporting long-term viability. The sensitivity analysis, conducted across multiple thresholds, demonstrated that while several configurations remained stable, higher thresholds led to more restrictive solutions. At 0.80, only two configurations remained, representing the most consistent pathways to sustained activity, reinforcing the importance of governance and transaction models in initiative sustainability.

**Conclusions:**

The analysis revealed a range of governance, ownership, business, and sustainability models, with no single structural configuration guaranteeing long-term viability. The findings suggest that governance and transaction mechanisms are particularly influential in sustaining initiatives, often compensating for the absence of strong business or sustainability models. The scope was limited to initiatives identified through available documentation and snowball sampling, and the results underscore the need for further research into the interplay between governance structures, financial models, and long-term sustainability in medical data sharing.

## Introduction

### Overview

As medical data have moved to being a solely digital artifact [1] and an increasing amount of health-related data has become available from noninstitutional sources [2], a large number of private businesses have emerged aiming to use these data and to profit from their use [3]. The inherently sensitive nature of medical data and the potential implications of it being misused or misappropriated mean that there is an increased need to constrain the use of this data from privacy, legal, and ethical standpoints [4]. These constraints, though, must be set against the potential of medical data to enable immense benefits in the areas of personal, public, and research-level health [5]. While government-funded or public initiatives to facilitate the use and reuse of medical data can play a role in leveraging secondary use of medical data for the public good, there is still a huge scope for benefit to be gained from allowing or encouraging private third parties to build for-profit businesses based on the consented use of personal medical information [6]. This then produces a natural tension between the benefit (both direct and indirect) of organizations obtaining medical data and associated permission to use it on the one side and a resistance, both on personal and jurisdictional levels, to sharing data on the other. Set alongside this is the need for such businesses to incentivize potential data subjects to consent to the use of their data and to do this in a way that is both ethically and legally sound [7].

Given the legal, ethical, and reputational risks and limitations associated with the use of medical data for profit-making means, the structure of the business models that enable this, and the means by which they navigate these risks, is of interest [8]. The sharing of medical data has the potential to transform health care, enabling better patient care, faster diagnoses, and more effective treatments [9]. However, it also raises important questions around privacy, ownership, and control. In recent years, there has been a proliferation of medical data sharing initiatives, from large-scale public-private partnerships to smaller-scale collaborations between health care providers and tech companies. While these initiatives hold promise for improving health care outcomes, they also raise important legal, ethical, and reputational concerns that must be carefully navigated. The structure of the business models underpinning these initiatives, and the measures taken to manage legal and ethical risks, therefore, warrants closer examination. To gauge the current landscape of medical data sharing initiatives, we have conducted a review, looking at existing medical data sharing initiatives and business models, their structure, and methods of incentivization. This paper presents the results of this survey.

This survey was conducted in the broader context of ongoing efforts to develop innovative and sustainable business models for the use of medical data. These efforts aim to balance the immense potential of medical data to improve health care outcomes with the critical need to address privacy, legal, and ethical concerns. By systematically reviewing and analyzing various medical data sharing initiatives, we look to inform the development of frameworks that ensure ethically sound and legally compliant use of medical data. The focus on blockchain technology reflects a growing interest in leveraging its capabilities to enhance data security and transparency, potentially addressing some of the key challenges in medical data sharing.

### Background

As health care data have become predominantly digitized [10,11], there is increasing recognition of the value of digital data in driving innovation in general areas of public health [12], including public responses to the recent COVID-19 epidemic [13] and specific research fields such as genomics [14], dementia [15], and diabetes [16], among others. Alongside the digitization of health care data at the source (eg, within health care institutions), there has been a rise in personal health monitoring and data collection through commercially available wearable devices [17,18]. The growth of informatics infrastructure necessary to process and transport data, enable data linkage across datasets [19], and manage consent and sharing preferences [20] has further enabled third-party entities to use medical data for both public and private use [21].

Given the sensitive and personal nature of medical data, there are additional requirements for data processing and handling, particularly by third parties [22]. Ethical considerations for the use of data [23] and moral concerns as to its use [24], often becoming more limited depending on the specific type of medical data (eg, that relating to sexual health [25,26], mean that, even in conjunction with general limitations on the use of personal data [27], the use of such data is inherently limited. These ethical considerations translate into more firmly enforced legislative constraints [28], placing additional constraints on business models that seek to leverage personal medical data for profit-making purposes [3].

One area of interest in the use of medical data is the emergence of blockchain technologies [29], which offer opportunities for actors in a business transaction to communicate, transact, and share information in an open, tamper-proof, and trustless manner [30]. Blockchain is the underlying technology initially used to implement the Bitcoin cryptocurrency [31]. A blockchain is a data structure that encodes a series of blocks of data in series, with each block containing a cryptographic reference to the previous block in the chain. Combined with a consensus mechanism, which is a protocol for determining among a distributed set of potentially untrustworthy peers which series of blocks is the currently accepted chain, blockchains offer a technological means of sharing canonical sets of information in a trustless manner across a wide range of participants [32]. Blockchain-based systems have been enhanced by the development and application of smart contract technologies, which allow the formation of blockchains to be controlled and enhanced through the use of programmatic functions that control the input and output of transactions and data on-chain [33]. Blockchain technology has been adopted across a wide range of industries [34]. The use of blockchain technologies in the context of medical data sharing initiatives potentially allows for the construction of systems that provide guaranteed openness of data use [29], removal of the need for trusted third parties to coordinate actions such as data linkage [35], and a native layer for offering, processing, and distributing tokens of incentivization [36]. The use of blockchain technology as the underlying mechanism for enabling the sharing and use of personal medical data by third parties is increasingly common at the present time [37].

In the era of data-driven health care, the growth in digital health information offers potential for improving patient outcomes and public health [38]. However, fragmented and siloed health data present significant challenges [39]. Data cooperatives address these challenges by fostering collective sharing, management, and governance of data among diverse stakeholders, including patients, health care providers, researchers, and institutions [40]. These cooperatives promote transparency, privacy, and informed consent, empowering individuals with greater control over their health information while adhering to regulatory and ethical standards [41]. Through collaboration and data pooling, data cooperatives bridge the gap between data availability and research, enabling comprehensive datasets for innovative medical research and evidence-based policymaking, driving improvements in patient outcomes and public health [42]. Data cooperatives differ from traditional data management approaches in several key ways. They operate on principles of inclusivity and collaboration, involving a wide range of stakeholders in the data sharing process. Patients have a central role, ensuring the protection of their privacy rights and determining access to their health information [43]. Advanced technologies and standardized protocols are used to ensure data interoperability, enhancing the quality and comprehensiveness of shared data [44]. Governance structures involve representatives from legal, ethical, and technical fields to develop policies that safeguard data integrity and security while fostering trust among participants [45].

In health care, data cooperatives can revolutionize the sharing, access, and use of sensitive medical information. By pooling diverse health datasets, cooperatives provide comprehensive views of patient medical histories, aiding health care professionals in making informed decisions about diagnosis, treatment, and care plans. Collaborative research efforts are facilitated by access to large and diverse datasets, promoting discoveries and advancements in medical science. Patients benefit from improved health outcomes by gaining control over their data and ensuring its protection [22]. Data cooperatives enhance population health initiatives by supporting data-driven policy decisions and public health interventions, advancing health care systems, and contributing to improved health outcomes on a broader scale. Despite their collaborative nature, viable business models are still needed to support the operation and sustainability of these cooperatives.

### The Civic Data Identity Partnership

The Civic Data Identity Partnership (CDIP) project aimed to develop a technical platform to manage access to health data. The primary objective was to create an infrastructure that would allow citizens to see, control, and provide consent for the usage of their health data in research and decision-making processes. One of the aims of CDIP was to explore the viability of business models that support the sustainability of the platform [46].

To achieve these aims, the project engaged in consultations with a diverse range of stakeholders, including citizens, clinicians, medical personnel, and data commissioners. These consultations were crucial in identifying the technical, legal, social, and ethical challenges associated with using the CDIP platform to enhance access and control over health data. The project aimed to understand the level of control individuals desired over their health information sharing and the data requirements of other parties. The collaborative efforts of the CDIP project provided insights into the complex landscape of health data management. Stakeholder consultations addressed technical intricacies, legal implications, societal concerns, and ethical considerations [46]. By incorporating these perspectives, the project aimed to design a platform that integrated access and control of health data, fostering transparency, privacy, and informed decision-making. Ultimately, the CDIP project sought to establish an infrastructure that met the preferences and requirements of citizens, clinicians, and other key stakeholders, enabling them to leverage health data while maintaining autonomy over its usage [47].

## Methods

### Aims and Objectives

This study aimed to examine a selected set of blockchain-based medical data sharing initiatives, analyzing their governance structures, incentive mechanisms, ownership models, business approaches, transaction mechanisms, and sustainability strategies. The objective was to identify common characteristics and contextual factors that may influence their development and persistence.

The specific objectives were:

1. To identify blockchain-based medical data sharing initiatives using a combination of academic literature review, targeted web searches, and expert consultations.
2. To categorize these initiatives based on predefined criteria, including organization type, governance structure, incentive mechanisms, ownership model, business model, transaction mechanisms, and sustainability strategies.
3. To assess the operational status of the identified initiatives, examining whether they have continued, evolved, or ceased operations.
4. To analyze emerging patterns in governance, incentives, and sustainability, providing insights into the challenges and factors influencing the persistence of these initiatives.

### Longitudinal Assessment of Identified Initiatives

In addition to categorizing and analyzing blockchain-based medical data sharing initiatives, the study included a longitudinal assessment to examine changes in their operational status. This involved tracking whether previously identified initiatives remained active and whether they had evolved in terms of governance, business strategies, or technical implementation. To conduct this assessment, publicly available sources—including websites, white papers, and social media updates—were reviewed, and project teams were contacted where possible to gather additional insights. The objective was to better understand the factors influencing the stability and evolution of these initiatives over time. By examining the characteristics and status of blockchain-based medical data sharing initiatives, this study aimed to contribute to ongoing discussions around their feasibility, governance, and sustainability. The findings provide insights for stakeholders considering the adoption of blockchain in medical data sharing and highlight areas for future research.

### Methodology

#### Overview

The study used a 2-stage methodology to examine blockchain-based medical data sharing initiatives. The first stage involved snowball sampling to identify initiatives with publicly available documentation, drawing from academic literature, online databases, industry reports, and directories of blockchain projects and startups. This approach ensured that selected initiatives had sufficient data for structured analysis.

The second stage involved systematic screening and classification, where initiatives were evaluated based on predefined criteria, including governance, incentives, ownership, business models, transaction mechanisms, and sustainability strategies. To identify structural patterns and relationships among these characteristics, the study applied qualitative comparative analysis (QCA). This method allowed for the systematic comparison of governance and sustainability models across initiatives, providing insights into factors influencing their operational status and persistence.

#### Snowball Sampling Methodology

Snowball sampling is a nonprobability sampling technique where existing study subjects recruit future subjects from among their acquaintances, creating a “chain referral” system. This method is particularly useful for accessing hard-to-reach or hidden populations by leveraging the social networks of initial participants [48]. Similarly, it can be applied to surveying literature and initiatives, where the initial set of identified sources and projects serves as the starting point, and the search is expanded by examining references within those sources and consulting with experts in the field. The initial set of initiatives identified through search terms served as the starting point for the snowballing approach, which was further expanded by examining references and consulting with experts.

The snowball sampling technique was chosen for this survey to capture a comprehensive range of medical data sharing initiatives, particularly those on the cutting edge or in experimental stages that may not have formal publications. Initially, relevant initiatives were identified through academic literature and web searches using the specified search terms. Subsequently, references within these sources were explored, and experts in the field were consulted to uncover additional initiatives. This approach was essential due to the dynamic nature of the field, where many projects, particularly those from startups and experimental ventures, may not be widely documented in formal literature but are actively discussed in online forums, social media, and industry reports. This iterative process allowed for the inclusion of diverse initiatives, ensuring a thorough and representative survey.

#### Search

To obtain a broad list of medical data research initiatives, a snowball sampling technique was used. The initial search was conducted in June 2022, and results were revisited in March 2024 for comparison. This search involved reviewing academic publications, reports, and relevant online sources using a wide range of search terms related to medical data sharing and blockchain technology. The following search terms were used: “medical data,” “healthcare data,” “clinical data,” “data sharing initiative,” “Data Coop,” “data bank,” “data trust,” “data brokerage,” “blockchain data sharing,” “DLT,” and “Distributed Ledger Technology.”

In addition to formal search terms, more informal terms and phrases were used for web-based and social media searches. These included keywords and phrases commonly used in industry discussions, online forums, and social media platforms, which helped in identifying initiatives that may not have formal academic publications. The informal search terms included blockchain health data, health data exchange, medical data blockchain project, digital health data sharing, health data startup, and blockchain health project.

Following the initial identification of initiatives through direct searching, the snowball sampling technique was applied by tracing references within the initial results. In academic literature, this involved 2 approaches: examining reference lists of relevant articles to find additional cited works and identifying new articles that cited these initial papers. For web-based and social media searches, discussions, and posts about identified initiatives were reviewed for mentions of other related projects, including links to comparable initiatives within forums and professional groups. Expert consultations further informed the process, providing insights into ongoing projects with available documentation. This iterative approach facilitated the identification of initiatives with sufficient publicly available information for structured classification and analysis, ensuring a relevant and analyzable dataset for QCA.

#### Screening

Each identified initiative was initially screened to confirm that it qualified as a medical data sharing initiative. Subsequently, they were categorized based on the following criteria: organization involved, initiative description, country of operation, blockchain-based technology, incentives for citizens, ownership, governance, business and transaction model, and sustainability. The initiatives were then screened to include only those projects that could be classified along these dimensions. The screening was conducted by 3 independent reviewers, with discrepancies resolved through discussion and consensus. This process ensured that the initiatives included had sufficient information available in these categories to conduct a comprehensive analysis.

1. Organization: Evaluated the mission and values of the organization behind the initiative.
2. Description: Assessed the scope and goals of the project.
3. Country-Based: Considered the geographical location and its potential impact.
4. Blockchain-Based: Evaluated the use of blockchain technology and its suitability for medical data.
5. Incentives for Citizens: Assessed the initiative’s approach to incentivizing data sharing among citizens, including data control and privacy.
6. Ownership: Evaluated the ownership structure, including single-entity or membership-based models.
7. Governance: Assessed the governance structure and the role of stakeholders.
8. Business Model: Evaluated revenue streams and funding sources.
9. Sustainability Model: Assessed the long-term sustainability strategy of the initiative.

This screening process allowed for a systematic evaluation, ensuring the inclusion of initiatives that met the inclusion criteria and could provide insights into medical data sharing initiatives.

#### Evaluation of Ongoing Project Activity

A longitudinal assessment was conducted to evaluate the operational status of identified initiatives. An initial dataset was compiled between 2020 and 2023 as part of the CDIP project [46]. A follow-up assessment was performed between December 2023 and March 2024 to determine whether initiatives remained active, had evolved, or had ceased operations.

To determine whether the identified initiatives were still operational, we used a multistep approach involving direct verification and indirect evidence collection. The steps included:

1. Official Websites and Social Media: We visited the official websites and social media pages (LinkedIn, Twitter/X, Facebook) of each project. Active projects typically have recent posts, updates, or blog entries. The presence of recent content was considered an indicator of ongoing activity.
2. News Articles and Press Releases: We searched for recent news articles or press releases about the projects. Projects that are still operational often appear in the news regarding new partnerships, product updates, or funding rounds. These sources provided additional confirmation of the projects’ statuses.
3. GitHub and Open Source Repositories: For open-source projects, we checked their GitHub repositories. We looked at the dates of the latest commits, pull requests, and issues. Active projects typically have recent activity in their repositories.
4. Industry Reports and Databases: We used industry databases such as Crunchbase, AngelList, and specialized blockchain directories to see if the projects were still listed as active. These platforms often provide updates on the operational status of companies.
5. Direct Inquiries: We sent inquiry emails to the contact information provided on the projects’ websites. Direct communication with project teams or representatives was used to obtain the most accurate and up-to-date information regarding the current status of the initiatives.
6. Community Forums and Discussion Boards: We monitored blockchain and tech community forums like Reddit, Bitcointalk, and industry-specific channels. Discussions and posts in these forums provided insights into whether the projects were still considered active by the community.

By using these methods, we were able to systematically verify the operational status of each project, allowing an assessment of their current activity levels.

#### Qualitative Comparative Analysis

##### Overview of QCA as an Analytical Method

QCA is a set-theoretic comparative method that allows for the systematic examination of multiple cases to identify patterns and relationships between conditions and outcomes [49]. Compared to traditional variable-based statistical methods, QCA is particularly well-suited for analyzing complex social and organizational phenomena where causation is often configurational rather than linear. It enables the identification of necessary and sufficient conditions for specific outcomes, making it a suitable approach for evaluating how different governance models, ownership structures, business models, and incentive mechanisms influence the persistence of blockchain-based medical data sharing initiatives.

QCA operates by assigning categorical values to case attributes and then applying a comparative logic to determine which configurations of conditions are consistently associated with an outcome of interest. This method has been widely used in political science, management research, and social sciences to identify structural patterns among cases with diverse characteristics [50].

##### Application of QCA

In this study, QCA was applied to analyze whether specific combinations of governance models, ownership structures, incentive mechanisms, business models, and sustainability strategies were systematically associated with the continued operation or cessation of blockchain-based medical data sharing initiatives. The analysis followed a structured four-step process:

1. Case Selection and Calibration: Each initiative was treated as a case and categorized according to predefined dimensions. Attributes were assigned multivalue categorical codes to capture variation across initiatives. The outcome variable—whether an initiative remained active or ceased operation—was also coded accordingly. Fuzzy-set calibration was applied, converting categorical values into membership scores between 0 and 1, ensuring the analysis could accommodate varying degrees of attribute presence.
2. Construction of a Truth Table: A truth table was generated, listing all unique combinations of conditions present in the dataset. Each configuration was mapped to the corresponding outcome status of the initiatives exhibiting that combination of attributes. This step allowed for an initial assessment of how different structural characteristics were associated with sustained activity and grouped cases with similar profiles.
3. Identification of Configurational Patterns: The truth table was minimized using the Quine-McCluskey algorithm, which systematically reduced the dataset to the simplest set of configurations that explained the observed outcomes. The minimization process identified necessary and sufficient conditions: necessary conditions appeared in all active initiatives, while sufficient conditions represented combinations of attributes that consistently led to an active status, even if no single factor was universally present.
4. Interpretation and Sensitivity Analysis: The minimized configurations were examined to identify distinct pathways leading to sustained initiative operation. The results demonstrated equifinality, meaning multiple different configurations could lead to the same outcome. Sensitivity analysis was conducted by adjusting the inclusion threshold for sufficiency, revealing how different conditions varied in their explanatory power across different levels of consistency.

This approach provided a systematic framework for assessing the structural factors influencing the viability of medical data sharing initiatives. The findings clarified how different combinations of governance, incentives, and sustainability mechanisms contributed to continued operation, offering insights into the conditions under which such initiatives remain active over time.

##### The Use of QCA

The use of QCA in this study is justified by the complex, multidimensional nature of blockchain-based medical data sharing initiatives, where governance structures, incentive mechanisms, and sustainability strategies interact in nonlinear ways. Unlike traditional regression-based approaches that assume additive relationships between independent variables, QCA allows for configurational causation, meaning that different pathways can lead to similar outcomes.

QCA provides a structured, comparative analysis of how blockchain-based initiatives are structured and which conditions are associated with long-term viability. This approach offers insights that can inform the design of future initiatives and policy frameworks aimed at sustaining blockchain-based medical data sharing models.

## Results

### List of Initiatives

Our search found 42 initiatives related to medical data sharing that were identified through the 2-stage methodology outlined in the previous section. Each initiative was systematically screened and analyzed along different dimensions to categorize them based on various characteristics. The resulting table provides an overview of the initiatives and their features, including information on their country of origin, the type of blockchain technology used, incentives for citizen participation, ownership structure, governance, business model, transaction model, sustainability, and whether they are public, private, or hybrid blockchains. A description of each initiative is provided in Table 1, highlighting key features and characteristics of each project.

**Table 1.** List of initiatives.

Organization	Description
e-Nome	Platform for consumers to control the use of their health and genomic data via blockchain technology.
Shivom	Blockchain-based genomics marketplace empowering individuals to control access to their genomic data.
Our data helps/Qntfy	Data donation for suicide prevention using social media and fitness data, powered by Qntfy.
EverGreen Life	App integrating GP^a^ records, health data, and wellness information for user control.
Medical Chain	Decentralized platform for secure, fast, and transparent exchange and usage of medical data using blockchain.
My Clinic	Tool for health care professionals to connect with patients and share health records securely.
MIDATA	Nonprofit Swiss cooperative for storing personal health data and enabling participation in app-based research.
HealthBank Coop	For-profit Swiss cooperative health data exchange platform with complete data ownership for users.
Serto (formerly uPort)	Identity and trust management platform for verified data exchange and decentralized identity protocols.
Savvy Coop	Patient-owned cooperative connecting innovators with patients and caregivers for insights and rewards.
Sync for Science	Collaboration among EHR^b^ vendors and NIH^c^ to support the Precision Medicine Initiative by sharing EHR data.
Salus Coop	Citizen-owned cooperative facilitating data sharing to accelerate health research and innovation.
Carbon Coop	Cooperative focused on reducing home carbon emissions using innovative energy efficiency solutions.
23andMe	Personal genomics company providing genetic testing services and enabling research participation.
DNAtix	Platform for anonymous and secure DNA testing and storage using blockchain technology.
Zenome	Russian biotech company using blockchain to empower consumers to control and sell their genomic data.
Nebula Genomics	Personal genomics company providing affordable whole genome sequencing with blockchain-enabled data privacy.
PokitDok	API^d^ platform for streamlining health care transactions and integrating real-time health insurance data.
Patientory	Platform for managing and analyzing population health data using blockchain technology.
Guardtime	Blockchain technology for security and scalability in various enterprise solutions, including Estonia’s e-government.
Gravitate Health	Digital health information project co-led by the University of Oslo and Pfizer, using open-source digital platforms.
Chronicled	Supply chain solution for the health care system using blockchain technology for trust and automation.
Gem	Blockchain identity platform for secure data sharing and identity management in health care.
Doc.AI	AI^e^ platform for health data collection and management, using blockchain for data security.
Iyro	Protocol for secure and private exchange of medical data using blockchain and proxy re-encryption.
Coral Health	Platform for secure sharing of medical records and personal health data using blockchain.
EncrypGen	Platform for secure exchange of genomic data for cryptocurrency tokens using blockchain.
Blockpharma	Blockchain solution for drug traceability and counterfeit detection.
BurstIQ, Inc	Blockchain-enabled data network solutions for health care, focusing on data sharing and monetization.
Bodyo	Telehealth devices and AI profiling for health monitoring and chronic disease prevention.
Exochain	Blockchain platform for secure patient data interaction in clinical trials and health care.
PharmaLedger	Blockchain-enabled health care solutions platform, supported by the Innovative Medicines Initiative.
Curisium/Healthverity	Platform for secure, privacy-compliant real-world data exchange in health care.
HealthCombix	Blockchain platform for patient data control, disease prediction, and decentralized payments.
SimplyVital Health	Platform for exchanging health data among medical providers.
Blockchain Health Co	Platform for direct sharing of medical information with researchers using blockchain.
Akiri	Software-defined network for secure and compliant health care data transport without data storage.
Embleema	Blockchain network for secure sharing of personal health records and participation in clinical trials.
Med.me	Blockchain-based platform for rapid exchange of electronic health records and medical data transactions.
Ethereum Health Wallet	Blockchain platform for storing and sharing medical records globally.
Healthbank Technologies	Direct-to-patient platform for health care providers to connect with patients for various services.
Solve.Care	Blockchain technology platform aimed at reducing health care administration costs and improving outcomes.

a GP: general practitioner.

b EHR: electronic health record.

c NIH: National Institutes of Health.

d API: application programming interface.

e AI: artificial intelligence.

### Case Selection and Calibration

#### Overview

We analyzed the selected set of blockchain-based medical data sharing initiatives, categorizing them across key dimensions relevant to their structure and sustainability. The analysis focused on governance models, ownership structures, business models, sustainability strategies, incentives for participation, blockchain adoption, and transaction mechanisms. These categories were selected based on their potential impact on the viability and persistence of initiatives.

Governance models were inferred where explicit information was unavailable, using related factors such as ownership type, decision-making structures, and regulatory compliance. Similarly, for other categories, when direct descriptions were not provided, classifications were assigned based on publicly available documentation, project descriptions, and associated operational characteristics. This approach ensured that initiatives were systematically categorized for comparative analysis while acknowledging variations in reporting detail.

#### Governance Model

##### Overview of Governance Structures Identified

Governance refers to the decision-making structures and oversight mechanisms used in medical data sharing initiatives. The analysis identified four primary governance models, with an additional category for cases where governance structures were unclear:

1. Board-Based Governance: Initiatives governed by a formal advisory board, ethics committee, or corporate governance structure. These models were common among private, for-profit initiatives where oversight was centralized within an organization. This classification was assigned when documentation referenced board decision-making, formal advisory roles, or corporate governance policies.
2. Regulation-Based Governance: Initiatives whose governance was defined primarily by compliance with legal or regulatory frameworks, such as the General Data Protection Regulation (GDPR). These were often government-backed or public-private partnerships, and initiatives mentioning strict compliance requirements or legal structures were assigned to this category.
3. Membership-Based Governance: Initiatives governed by their participants, such as patient-led or cooperative models. These initiatives often had democratic decision-making structures, where users or stakeholders collectively determined policies. This classification was used when initiatives described member voting rights, cooperative ownership, or user-led decision-making.
4. User-Regulated Governance: Initiatives where governance was decentralized, with individual users retaining control over their own data. These models emphasized self-sovereign identity frameworks or direct user participation in access control. This category was assigned where initiatives explicitly stated that users determined governance decisions or when decentralized, self-regulated platforms were described.
5. No Clear Governance: Initiatives for which governance structures were not documented or could not be inferred from available information.

This classification allowed for a structured comparison of governance mechanisms across initiatives, facilitating the identification of patterns in oversight structures and their relationship to long-term viability.

##### Criteria for Classification

Governance was classified based on how decision-making authority and oversight mechanisms were structured within each initiative. Given the lack of explicit governance descriptions in many cases, classification was inferred using related factors such as ownership type, decision-making structures, regulatory references, and operational frameworks. The following criteria were applied:

1. Board-Based Governance: Assigned when documentation referenced a board of directors, advisory board, or corporate governance policies. Typically found in private for-profit initiatives with centralized oversight.
2. Regulation-Based Governance: Applied to initiatives explicitly governed by external legal or regulatory frameworks such as GDPR compliance. Often observed in government-led or public-private models.
3. Membership-Based Governance: Identified when initiatives had collective governance by members, including cooperative ownership structures or voting mechanisms.
4. User-Regulated Governance: Assigned to initiatives where governance was decentralized, and individuals retained direct control over their own data and permissions.
5. No Clear Governance: Used when no governance information was available or could not be inferred from ownership or decision-making structures.

##### Distribution of Governance Models

Board-based governance was the most frequently observed model, particularly among private-sector initiatives with venture capital backing, where centralized oversight structures were well-defined. Regulation-based governance was present in government-backed projects and public-private partnerships, where compliance requirements shaped governance frameworks. Membership-based governance was less common but found in cooperative initiatives emphasizing patient or citizen participation. User-regulated governance was primarily associated with decentralized, blockchain-driven initiatives, particularly those leveraging self-sovereign identity models. Some initiatives lacked clear governance documentation, making classification difficult in cases where decision-making structures were not explicitly defined.

### Ownership Structures

#### Overview of Ownership Structures Identified

Ownership structures define who controls an initiative and how decisions are made regarding data access, governance, and sustainability. The following six ownership models were identified:

1. Private Ownership: Initiatives controlled by a single private entity, such as startups or corporations. These organizations typically operate on a for-profit basis, with centralized decision-making.
2. Public-Private Partnership: Collaborative models where government entities work with private sector organizations. These initiatives often balance public-sector oversight with private-sector innovation and funding.
3. Government-Owned: Fully state-controlled initiatives established to support public health infrastructure, regulatory frameworks, or government-led research projects.
4. Membership-Owned (Patients/Citizens): Initiatives where individuals contributing data collectively own and participate in decision-making. These models often emphasize user rights, transparency, and democratic governance.
5. Cooperative Membership: Formal cooperative organizations where control and decision-making are distributed among a legally recognized group of members, often structured around collective ownership principles.
6. No Clear Ownership: Initiatives where ownership details were unavailable, unclear, or not explicitly documented. These cases included decentralized projects or those with insufficient public records.

#### Criteria for Classification

Ownership structures were categorized based on the legal and organizational control of each initiative. Where ownership was not explicitly stated, classification was inferred from governance models, business structures, and stakeholder involvement. The following categories were used:

1. Private ownership was assigned to initiatives owned and controlled by a single private entity, such as startups or corporations. These initiatives typically operated on a for-profit model, with decision-making centralized within the organization.
2. Public-private partnership applied to initiatives where government entities collaborated with private sector stakeholders. These initiatives often combined public-sector oversight with private-sector innovation.
3. Government-owned initiatives were those fully controlled and operated by government agencies, typically established to support public health infrastructure or regulatory compliance.
4. Membership-owned (patients/citizens) initiatives were owned and governed by their participants, allowing individuals contributing data to have a direct role in decision-making. These models often aligned with cooperative governance structures.
5. Cooperative membership was assigned to initiatives collectively owned by a cooperative entity, where decision-making was distributed among members. Unlike membership-owned initiatives, cooperatives functioned as formal legal entities.
6. No clear ownership was assigned when no ownership structure was documented, and no inference could be drawn from related governance or business structures.

#### Distribution of Ownership Models

Private ownership was the most common structure, particularly among blockchain-based initiatives developed by startups or corporate entities. Public-private partnerships were relatively rare but present in projects that combined public health objectives with private-sector innovation. Government ownership was observed in select national health initiatives but was not a dominant model. Membership-owned initiatives were primarily seen in patient-driven projects that emphasized collective governance, while cooperative membership structures were identified in a smaller subset of initiatives explicitly structured as cooperatives. Some initiatives did not provide sufficient documentation to determine ownership, leading to classification under no clear ownership.

### Business Model Structures

#### Overview of Business Model Structures Identified

Business models refer to the revenue-generating and operational frameworks used by medical data sharing initiatives. The analysis identified five primary business model structures, with an additional category for cases where the business model was unclear:

1. For-Profit Models: Initiatives that generate revenue through commercial activities such as subscription fees, data access sales, or transaction-based charges. This category was further divided into:
  - Fee or Subscription-Based Models: Users pay a recurring or one-time fee for access to services or data platforms.
  - Data Resale or Reuse-Based Models: Revenue is generated by selling or repurposing user data for research or commercial use.
  - Venture Capital-Backed Models: Initiatives funded by external investment, often prioritizing growth and scalability.
  - General For-Profit Models: Commercial initiatives that do not fit neatly into the above subcategories but generate revenue through various business activities.
2. Not-for-Profit Models: Initiatives that focus on public benefit rather than financial gain. These are often supported through grants, partnerships, or government funding and typically aim to improve health care, support research, or provide public health benefits.
3. Cooperative Models: Member-owned initiatives where participants collectively own and govern the organization. These models prioritize ethical data usage, community governance, and equitable distribution of benefits.
4. Membership Reimbursement Models: Initiatives where participants receive direct financial incentives or token-based compensation for contributing data. These models encourage participation by offering rewards or benefits tied to data sharing.
5. Unknown or Unclear Models: Initiatives where the business model was not explicitly documented or could not be inferred from available information.

This classification enabled a structured comparison of revenue strategies across initiatives, helping to identify patterns in financial sustainability and economic incentives.

#### Criteria for Classification

Business models were classified based on how initiatives generated revenue and sustained operations. Given the lack of explicit financial disclosures in some cases, classification was inferred using related factors such as pricing structures, funding sources, and service models. The following criteria were applied:

1. For-Profit Models: Assigned when initiatives explicitly charged for services, sold data access, or referenced investor-backed growth strategies.
  - Fee or Subscription-Based: Used when initiatives charged users a recurring or one-time fee.
  - Data Resale or Reuse-Based: Applied when initiatives monetized user data through resale or licensing agreements.
  - Venture Capital-Backed: Identified when initiatives referenced venture capital funding or external investment rounds.
  - General For-Profit: Used for commercially driven initiatives that did not clearly fit into other subcategories.
2. Not-for-Profit Models: Applied to initiatives that identified themselves as nonprofit organizations or relied primarily on grants, donations, or public funding.
3. Cooperative Models: Assigned when initiatives described collective governance structures where members had ownership stakes and participated in decision-making.
4. Membership Reimbursement Models: Used when initiatives provided direct incentives (eg, financial rewards, cryptocurrency, or tokens) for data sharing.
5. Unknown or Unclear Models: Assigned when no clear business model was documented or when financial sustainability mechanisms could not be determined.

#### Distribution of Business Models

For-profit models were the most common, particularly among private companies leveraging subscription fees, data monetization, or transaction-based revenue streams. Many of these initiatives were venture-backed, suggesting an emphasis on scalability and commercialization. Not-for-profit models were observed in research-driven projects and public-sector initiatives where financial sustainability relied on grants or government support. Cooperative models were less frequent but represented a distinct approach emphasizing member ownership and democratic decision-making. Membership reimbursement models were primarily found in blockchain-based initiatives where participants were incentivized through tokens or direct financial compensation. Some initiatives lacked clear business model documentation, making classification challenging where revenue mechanisms were not explicitly defined.

### Blockchain Usage

#### Overview

The analysis of blockchain use within medical data sharing initiatives gave three categories:

1. Blockchain-Based Initiatives: These initiatives use blockchain technology to enhance data security, transparency, and patient control. By leveraging blockchain’s decentralized ledger, they aim to ensure data integrity and facilitate secure sharing among stakeholders. Examples include e-Nome, Shivom, Medical Chain, and My Clinic.
2. Non-Blockchain-Based Initiatives: These initiatives use traditional centralized systems for data management and sharing. They may focus on interoperability and data exchange without incorporating blockchain technology. Examples include Savvy Coop, Sync for Science, Carbon Coop, and 23andMe.
3. Unknown Blockchain Usage: For some initiatives, the adoption of blockchain technology is unclear or not specified in available documentation. Examples include Our Data Helps/Qntfy, EverGreen Life, MIDATA, and Salus Coop.

#### Criteria for Classification

A project was classified as a blockchain-based project if it used blockchain technology as a core component of its architecture or if it claimed to use blockchain in its documentation or website. Projects that mentioned blockchain in passing or only used it for peripheral purposes, such as noncore payments functionality (eg, as an alternative currency for paying for goods or membership, as opposed to a direct reimbursement mechanism), were not classified as blockchain-based projects for the purposes of this study. The breakdown by blockchain usage is shown in the Truth Table section.

#### Distribution of Blockchain Usage

Most initiatives analyzed were blockchain-based, given the focus of our search methodology and reflecting a growing trend toward adopting decentralized technologies in the field of medical data sharing. These initiatives often highlight enhanced security, patient empowerment, and improved data interoperability as key benefits of blockchain integration. Non–blockchain-based initiatives continue to operate using established centralized systems, focusing on traditional methods of data exchange and management. A subset of initiatives lacked sufficient information to determine their use of blockchain technology, indicating a need for greater transparency in their operational disclosures.

### Incentive Models

#### Overview of Incentive Models Identified

Incentives refer to the motivations that drive individuals and organizations to participate in medical data sharing initiatives. The analysis identified 5 primary incentive models, which account for both direct and indirect benefits to participants. Some initiatives use multiple incentive models, combining elements such as financial compensation with altruistic motivations. The identified categories are:

1. Self-Interest: Participants are motivated by personal benefits such as improved health care access, personalized insights, or streamlined administrative processes. These initiatives provide users with direct advantages, such as access to health records, genetic analysis, or medical consultations.
2. Altruism: Participation is driven by a desire to contribute to the broader public good, such as supporting medical research or advancing health care innovation. These initiatives often emphasize collective benefits over individual gains.
3. Indirect Financial Incentives: Participants expect potential future financial gains, such as improved market positioning, enhanced reputation, or cost savings through participation. These initiatives may not offer direct financial compensation but provide economic advantages over time.
4. Direct Financial Incentives: Participants receive immediate financial compensation for contributing data, such as payments, tokens, or vouchers. This model is often seen in initiatives that monetize data access or participation in research.
5. Non–citizen-Based Incentives: Benefits are directed toward broader community outcomes rather than individual participants. These include initiatives that improve public health, enhance health care systems, or provide large-scale infrastructure benefits.

Many initiatives blend multiple incentive structures, allowing individuals and organizations to participate for different reasons. For example, some for-profit initiatives incentivize user participation through direct financial rewards while also positioning themselves as contributors to health care research. Others rely on a combination of self-interest and altruism to attract participants.

#### Criteria for Classification

Initiatives were classified based on how they motivate participation, as inferred from available documentation. The following criteria were applied:

1. Self-Interest: Assigned when initiatives provided personal benefits such as access to health data, enhanced health care services, or convenience-based incentives.
2. Altruism: Applied to initiatives where individuals contributed data or participated in research with no expectation of personal gain.
3. Indirect Financial Incentives: Used when future financial benefits were implied, such as improving market standing or gaining access to health care services that could lower costs over time.
4. Direct Financial Incentives: Assigned when participants received explicit monetary compensation or exchangeable tokens in return for their participation.
5. Non–citizen-Based Incentives: Used for initiatives that emphasized benefits to society, health care providers, or broader community health outcomes rather than individual rewards.

In cases where an initiative aligned with multiple incentive models, the primary motivation identified in its operational structure was used for classification.

#### Distribution of Incentive Models

Self-interest was the most commonly observed incentive model, particularly among initiatives offering health insights, access to medical records, or streamlined services. Altruism was also prevalent, especially in research-oriented initiatives where users contributed data without expecting financial returns.

Direct financial incentives were primarily associated with for-profit initiatives using tokenized compensation models or offering payments for data sharing. Indirect financial incentives were seen in initiatives that provided long-term economic benefits rather than immediate compensation.

Non–citizen-based incentives were less frequent but were present in initiatives aiming to improve public health outcomes, reduce systemic inefficiencies, or contribute to health care policy development. Many initiatives combined multiple incentive models, reflecting the complexity of participant motivations in the medical data sharing ecosystem.

### Sustainability Models

#### Overview of Sustainability Models Identified

The analysis of long-term business sustainability revealed that initiatives mainly relied on six different models to generate revenue and ensure operational longevity. Some initiatives combined multiple models to diversify income streams and reduce financial risk. The sustainability models identified are:

1. Membership Fee-Based: Initiatives charge recurring fees from members to cover operational costs. This model provides a predictable revenue stream and is commonly used by cooperatives or community-driven platforms.
2. Selling Data Access to Third Parties: Revenue is generated by granting access to medical data for research or commercial purposes. This model is often used by for-profit companies engaging with pharmaceutical firms, public health bodies, or academic researchers.
3. Selling Access to Users: Initiatives generate revenue by selling access to data-related services directly to individuals. This may involve subscriptions for premium services, one-time purchases, or pay-as-you-go models.
4. Further Investment: These initiatives rely on external funding sources such as venture capital, grants, or public investment to sustain their operations and drive growth. This model is common among startups and research-driven projects.
5. Software-as-a-Service (SaaS): Revenue is derived from subscription-based access to data platforms, analytical tools, or storage solutions. This model is typically used by technology-driven initiatives offering data integration or analytics services.
6. Unknown: Some initiatives did not clearly specify their revenue model or sustainability strategy, making it difficult to classify them.

#### Criteria for Classification

Sustainability models were classified based on the primary revenue mechanism described by each initiative. When explicit information was unavailable, classification was inferred from related indicators such as business model structure, funding sources, and platform offerings. The following criteria were applied:

1. Membership Fee-Based: Assigned to initiatives that charge individual or institutional users recurring fees for access.
2. Selling Data Access to Third Parties: Applied to initiatives where revenue is primarily generated through selling data to external organizations.
3. Selling Access to Users: Used for initiatives where users pay directly for services, reports, or data-related tools.
4. Further Investment: Identified in cases where venture capital, grants, or other funding mechanisms were the primary source of sustainability.
5. Software-as-a-Service (SaaS): Assigned to platforms offering subscription-based access to tools, data storage, or analysis services.
6. Unknown: Used when sustainability strategies were not explicitly documented or could not be inferred from available information.

#### Distribution of Sustainability Models

The distribution of sustainability models varied across initiatives, reflecting differences in business objectives, funding structures, and target users. Many for-profit initiatives relied on selling access to users, often through subscription-based services or premium data offerings, ensuring a continuous revenue stream. Others focused on selling data access to third parties, particularly in genomic and health data sharing platforms, where anonymized datasets were monetized for research and commercial applications. Several initiatives, especially cooperatives and patient-led organizations, adopted a membership fee-based model to maintain operations through recurring contributions from their members. Venture capital and other forms of external investment were a key source of sustainability for startups and research-driven initiatives, particularly those in early development stages. The Software-as-a-Service (SaaS) model was used by digital health platforms offering analytical tools or secure data storage on a subscription basis. However, a number of initiatives lacked clearly defined sustainability strategies, particularly among research collaborations and recently launched projects, indicating potential uncertainty in long-term financial viability.

### Transaction Models

#### Overview of Transaction Models Identified

Transaction models refer to the mechanisms by which initiatives facilitate exchanges of value, including payments for access, incentives for data sharing, and internal economic systems. The analysis identified five primary transaction models, with an additional category for cases where transaction structures were unclear:

1. Direct Monetary Transactions: Initiatives where users make direct payments to access data or services. This model provides immediate financial returns but may limit accessibility for individuals or organizations with fewer resources.
2. Token-Based Issuance and Sales (Single-Token Models): Initiatives using a single cryptocurrency token for internal transactions. These tokens may serve multiple purposes, including payments, incentives, or governance.
3. Dual Token Mechanism: Initiatives that use two distinct tokens, typically separating governance from utility transactions. This approach helps distinguish between platform operations and broader ecosystem participation.
4. Reward-Based Systems: Initiatives where participants receive incentives in the form of points, credits, or tokens for sharing data or engaging with the platform. This model is commonly used in research-driven initiatives seeking to encourage participation.
5. Cryptocurrency Payments: Initiatives that facilitate transactions using established cryptocurrencies such as Bitcoin or Ethereum. This model provides interoperability with external blockchain networks.
6. No Direct Payment: Initiatives where services or data are provided freely without requiring payment from users. These models often rely on alternative funding sources such as grants, partnerships, or donations.
7. No Clear Transaction Model: Initiatives for which transaction models were not explicitly documented or could not be inferred from available information.

This classification provides a structured framework for understanding the financial mechanisms that underpin data sharing initiatives and their implications for accessibility, participation, and sustainability.

#### Criteria for Classification

Transaction models were classified based on the primary method used to facilitate exchanges within each initiative. Given that some initiatives did not explicitly define their transaction models, classification was inferred based on references to payment structures, tokenized economies, or incentive systems. The following criteria were applied:

1. Direct Monetary Transactions: Assigned when initiatives specified user payments for services or data access, either as a one-time purchase or as a recurring subscription.
2. Token-Based Issuance and Sales (Single-Token Models): Applied to initiatives where a single cryptocurrency token was used for internal transactions, including payments or incentives.
3. Dual Token Mechanism: Identified in cases where initiatives used two separate tokens for different functions, typically governance and utility.
4. Reward-Based Systems: Used for initiatives that provided nonmonetary rewards, such as points or internal tokens, for participation or data sharing.
5. Cryptocurrency Payments: Applied when initiatives enabled transactions using standard cryptocurrencies rather than internal tokens.
6. No Direct Payment: Assigned when initiatives explicitly stated that data access or services were provided without direct payments from users.
7. No Clear Transaction Model: Used when no information on financial transactions was available or could not be inferred from the initiative’s structure.

This structured classification enabled a comparative analysis of how initiatives facilitated financial exchanges, highlighting different approaches to incentivization and economic sustainability.

#### Distribution of Transaction Models

Token-based systems were the most frequently observed transaction model, particularly in blockchain-based initiatives, where single or dual-token economies enabled internal transactions and incentivization mechanisms. Some initiatives relied on direct monetary transactions, particularly those offering commercial services or premium user features. Reward-based systems were implemented in research-driven initiatives, often as a means to encourage participation without requiring direct financial contributions. Cryptocurrency payments were less common but appeared in select initiatives that sought interoperability with external blockchain networks. A subset of projects provided services without direct payments, often relying on external funding sources. Finally, some initiatives lacked clear documentation of their transaction structures, making classification difficult in cases where financial models were not explicitly defined.

### Comparison to Initial Survey

#### Overview

The CDIP project ran between 2020 and 2023. An initial comprehensive review was conducted at the inception of the CDIP project to assess the landscape of health data initiatives and collaborative projects. This review served as a benchmark to understand the existing data sharing ecosystem and the potential impact of data cooperatives on the health care domain. At the conclusion of the CDIP project, a follow-up review was performed to evaluate the evolution of these projects over the course of the initiative.

#### Results of Project Activity Assessment

Following the reanalysis conducted from December 2023 to March 2024, we assessed the operational status of the 42 identified medical data sharing initiatives. Our findings are as follows: (1) out of the 42 projects, 28 projects are still active, and (2) 14 projects have become inactive.

These results provide insights into the current landscape of medical data sharing initiatives. The assessment highlights the dynamic nature of the field, with several projects ceasing operations over time. Understanding the factors contributing to the success or failure of these initiatives is important for future developments in the sector. The analysis will look at the continuation rates of the projects in the context of the categories used to screen the projects in the first phase of the analysis, such as incentive models, governance structures, ownership models, and sustainability strategies. This will help identify patterns and insights that can inform the design, implementation, and business approach of medical data sharing frameworks.

The projects have been categorized by their current status and are listed in Table 2.

**Table 2.** Projects by current status.

Status	Projects
Active	EverGreen Life, Medical Chain, My Clinic, MIDATA, Serto (formerly uPort), Savvy Coop, Sync for Science, Salus Coop, Carbon Coop, 23andMe, DNAtix, Nebula Genomics, Patientory, Guardtime, Gravitate Health, Chronicled, Gem, Iyro, EncrypGen, Blockpharma, BurstIQ.inc, Bodyo, PharmaLedger, Curisium/Healthverity, Med.me, Healthbank Technologies, Embleema, and Solve.Car
Inactive	e-Nome, Shivom, Our data helps/Qntfy, HealthBank Coop, PokitDok, Zenome, Doc.AI, Coral Health, Exochain, HealthCombix, SimplyVital Health, Blockchain Health Co, Akiri, Ethereum, and Health Walle

### Analysis of Results: Truth Table

#### Overview

A truth table was constructed to systematically represent the relationship between selected conditions and the observed status of each initiative in the follow-up assessment. The table organizes initiatives according to their characteristics, allowing for a structured comparison of configurations and outcomes.

The conditions included in the truth table were derived from the analysis of initiatives and categorized initiative structure and function. These conditions included ownership model, governance model, business model, transaction model, use of blockchain, incentive model, and sustainability approach. Each initiative was assigned values based on its classification in these categories. The outcome variable recorded the initiative’s status at follow-up, distinguishing between those that remained active or ceased operation.

The table allowed for an analysis of patterns among initiatives with similar configurations, supporting the identification of potential associations between specific conditions and initiative status. The structured format facilitated the next stage of analysis, where these patterns were further examined to assess whether particular combinations of conditions corresponded with different outcomes.

The analysis used fuzzy-set qualitative comparative analysis (fsQCA) to account for the varying degrees of membership within each condition. Unlike crisp-set QCA, which requires binary categorization, or multivalue qualitative comparative analysis (mvQCA), which assigns discrete categorical values, fsQCA enables conditions to take on continuous values between 0 and 1, allowing for a more nuanced representation of the initiatives. This approach was appropriate given the complexity of the dataset, where governance structures, ownership models, transaction mechanisms, and sustainability strategies exhibited varying levels of adherence to predefined categories rather than fitting into strictly distinct groups. This facilitated the identification of patterns in how different degrees of condition membership were associated with the observed outcomes, rather than limiting the analysis to rigid categorical distinctions.

#### Truth Table

#### Truth Table Representation

The truth table presents the data in a structured format to facilitate the identification of configurational patterns in the next stage of the analysis (Table 3). Each initiative is represented as a case, with categorical numerical values assigned to each variable (Textbox 1). These variables include ownership model, governance model, business model, blockchain usage, incentive model, sustainability model, transaction model, and the status of the initiative at the follow-up assessment.

**Table 3.** Truth table of analysis.

Organization	Ownership model	Governance model	Business model	Transaction model	Blockchain based	Incentive model	Sustainability model	Status
e-Nome	1	5	5	7	1	5	6	0
Shivom	1	5	1	2	1	4	3	0
Our data helps/Qntfy	1	5	2	6	3	2	3	0
EverGreen Life	1	2	1	3	3	1	3	1
Medical Chain	1	5	1	2	1	3	3	1
My Clinic	1	5	1	3	1	1	1	1
MIDATA	3	3	3	5	3	2	1	1
HealthBank Coop	3	3	3	2	1	4	4	0
Serto (formerly uPort)	1	4	1	5	1	1	3	1
Savvy Coop	3	3	3	5	3	4	4	1
Sync for Science	2	2	2	6	3	2	6	1
Salus Coop	3	3	3	5	3	2	6	1
Carbon Coop	3	3	3	5	3	2	6	1
23andMe	1	2	1	3	3	1	4	1
DNAtix	1	5	1	2	1	1	3	1
Zenome	1	5	1	7	1	4	3	0
Nebula Genomics	1	5	1	3	1	3	3	1
PokitDok	1	5	1	7	1	5	6	0
Patientory	1	5	1	2	1	1	3	1
Guardtime	1	5	1	7	1	5	6	1
Gravitate Health	2	2	2	6	1	2	6	1
Chronicled	1	5	1	7	1	5	6	1
Gem	1	5	1	6	1	1	6	1
Doc.AI	1	5	1	4	1	3	6	0
Iyro	1	5	1	4	1	3	6	1
Coral Health	1	5	1	5	1	1	6	0
EncrypGen	1	5	1	2	1	3	6	1
Blockpharma	1	5	1	7	1	5	6	1
BurstIQ.inc	1	5	1	2	1	3	6	1
Bodyo	1	5	1	5	1	3	6	1
Exochain	1	5	1	2	1	4	6	0
PharmaLedger	2	2	2	6	1	2	6	1
Curisium/Healthverity	1	5	1	5	1	5	6	1
HealthCombix	1	5	1	5	1	1	6	0
SimplyVital Health	1	5	1	7	1	5	6	0
Blockchain Health Co	1	5	1	7	1	5	6	0
Akiri	1	1	1	5	1	1	6	0
Embleema	1	5	1	2	1	4	6	1
Med.me	1	5	1	2	1	3	6	1
Ethereum Health Wallet	1	5	1	7	1	5	6	0
Healthbank Technologies	1	5	1	7	1	5	6	1
Solve.Care	1	5	1	3	1	3	6	1

Textbox 1. **Key for Table 3.**

**Ownership model**

1=Private business
2=Private business (investor backed)
3=Cooperative
4=Public-private
5=Unknown

**Governance model**

1=Board-based governance
2=Regulation-based governance
3=Membership-based governance
4=User-regulated governance
5=No clear governance

**Business model**

1=For-profit
2=Not-for-profit
3=Cooperative
4=Membership reimbursement
5=Unknown

**Blockchain usage**

1=Yes
2=No
3=Unknown

**Incentive model**

1=Self-interest
2=Altruism
3=Indirect financial incentives
4=Direct financial incentives
5=Non–citizen-based incentives
6=Unknown

**Sustainability model**

1=Membership fee-based
2=Selling data access to third parties
3=Selling access to users
4=Further investment
5=Software-as-a-service
6=Unknown

**Transaction model**

1=Direct monetary transactions
2=Token-based issuance and sales (single token)
3=Dual token mechanism
4=Reward-based systems
5=Cryptocurrency payments
6=No direct payment
7=Unknown

**Status**

1=Active
2=Inactive

This representation allows for systematic comparison of the initiatives, supporting the identification of configurations associated with initiative success or inactivity. The encoded values provide a standardized way to analyze the relationships between different attributes and their potential influence on outcomes.

### Identification of Configurational Patterns

The analysis of configurational patterns was conducted using the QCA package in R (The R Foundation), which provides a well-documented and widely used framework for performing QCA [51]. Specifically, fsQCA was applied to analyze configurations of conditions leading to the observed outcomes [52]. Unlike crisp-set QCA, which requires binary presence or absence of conditions, or mvQCA, which assigns discrete categorical values, fsQCA allows for graded membership scores between 0 and 1. This flexibility made it well-suited for capturing the continuous nature of governance models, transaction mechanisms, incentive structures, and ownership types observed in the dataset.

The minimization process was conducted using the Quine-McCluskey algorithm [53], which systematically reduces the truth table into a set of minimal sufficient conditions by identifying prime implicants—combinations of conditions that consistently lead to the outcome. This method provides an exhaustive and deterministic approach to simplification, contrasting with heuristic-based solutions that rely on approximation.

A central consideration in fsQCA is equifinality, the principle that multiple distinct configurations can lead to the same outcome. By applying fsQCA, the analysis accounted for the possibility that different combinations of governance models, transaction mechanisms, incentive structures, and ownership types could all contribute to similar sustainability or operational outcomes. This approach enabled a comparative yet flexible assessment of how initiatives maintain viability over time.

To assess the robustness of the findings, a sensitivity analysis was conducted by systematically varying the inclusion threshold, which determines the minimum consistency score required for a configuration to be considered sufficient for the outcome. The analysis tested inclusion thresholds between 0.60 and 0.80, examining whether the identified configurations remained stable or changed across different threshold settings. This step helped evaluate the reliability of the results and detect marginal cases that may influence the solutions.

The analysis was implemented in R, using scripts that preprocessed the data, calibrated conditions, constructed the truth table, and performed the minimization process. The resulting configurations were analyzed to provide insight into the structural pathways associated with initiative status in the follow-up assessment.

### Results of Configurational Analysis

#### Overview

The analysis examined the configurations of conditions that contributed to the sustainability of initiatives using fsQCA. This approach allowed for the identification of multiple pathways leading to sustained or inactive initiatives, rather than assuming a single causal relationship. The evaluation incorporated key attributes, including ownership models, governance structures, business models, incentive mechanisms, transaction models, and sustainability strategies.

A base model solution was first constructed using a consistency threshold of 0.65, providing an initial set of configurations that explained initiative outcomes. To assess the robustness of these findings, a sensitivity analysis was conducted by varying the consistency threshold across a range from 0.60 to 0.80. This iterative process tested the stability of the identified solutions and ensured that the results were not unduly dependent on a specific threshold choice.

By systematically analyzing the different conditions and their interactions, the study identified distinct structural configurations that were associated with sustained initiative activity. The results provided insight into how different combinations of governance, financial, and incentive models contributed to long-term viability, offering a comparative perspective on the factors influencing initiative success.

#### Base Model Solution

The base model, run at an inclusion threshold of 0.65, identified multiple configurations of conditions that were associated with the continued activity of initiatives. The minimization process resulted in two primary solutions (M1 and M2), each representing different pathways leading to sustained initiative status.

##### Minimized Configurations

The following configurations were identified as sufficient conditions for the outcome of sustained initiative activity (Y):

M1: ¬O * ¬B * ¬I * ¬T + ¬O * ¬G * ¬B * ¬T * ¬S

+ O * ¬G * B * ¬I * ¬S + O * ¬B * ¬I * T * S

+ G * ¬B * ¬I * ¬T * ¬S + (¬O * G * ¬B * ¬I * S) → Y

M2: ¬O * ¬B * ¬I * ¬T + ¬O * ¬G * ¬B * ¬T * ¬S

+ O * ¬G * B * ¬I * ¬S + O * ¬B * ¬I * T * S

+ G * ¬B * ¬I * ¬T * ¬S + (G * ¬B * ¬I * T * S) → Y

where O=ownership model, G=governance model, B=business model, I=incentive model, T=transaction model, S=sustainability model, and ¬ (negation) indicates the absence of the condition.

These minimized configurations presented in the base model represent combinations of conditions that are sufficient for an initiative to remain active. Each equation outlines a distinct pathway through which different structural elements contribute to sustained initiative status.

The expressions use logical conjunctions (“*”) to indicate that multiple conditions must co-occur, while the negation symbol (¬) denotes the absence of a given condition. The addition symbol (“+”) signifies alternative pathways—meaning that different sets of conditions can independently lead to the same outcome (sustained activity, Y).

##### Interpretation of Base Model Results

The interpretations of the base model results are as follows:

1. Multiple Viable Pathways: The results confirm equifinality, meaning that multiple distinct configurations can lead to the same outcome of sustained activity.
2. Role of Ownership and Governance: Initiatives that lacked a strong ownership structure (¬O) but had specific governance structures (¬G) were among those with sustained activity, suggesting that alternative governance mechanisms may compensate for weaker ownership models.
3. Business and Transaction Models: The absence of a defined business model (¬B) in multiple configurations indicates that some initiatives sustain themselves without a strong commercial model, possibly through alternative funding or cooperative structures.
4. Sustainability and Incentives: Some pathways include the presence of a sustainability model (S) and financial incentives (I), while others suggest that initiatives without clear incentive models (¬I) can still persist, particularly if they incorporate effective governance and transaction mechanisms.
5. Alternative Configurations: The presence of G * ¬B * ¬I * ¬T * ¬S suggests that some governance-led models can sustain without direct financial incentives or sustainability planning.

### Coverage and Consistency

#### Overview

The coverage table (Table 4) presents key metrics for the identified solutions (M1 and M2) that explain sustained initiative activity. The inclusion score (inclS) measures the consistency of each solution in explaining the observed outcomes. The proportional reduction in inconsistency further refines this by considering how well the solution differentiates between positive and negative cases. Solution coverage indicates the proportion of active initiatives explained by each solution. Since both solutions overlap significantly in the cases they cover, no unique coverage is present, meaning neither solution explains a distinct subset of initiatives that the other does not.

**Table 4.** Coverage and consistency.

Solution	Inclusion score (inclS)	Proportional reduction in inconsistency (PRI)	Solution coverage (covS)
M1	0.774	0.774	0.44
M2	0.784	0.784	0.447

The results confirm equifinality, showing that multiple distinct pathways can lead to sustained initiative activity. The high inclusion scores (≥0.77) indicate strong consistency, suggesting that these configurations reliably capture the factors associated with continued operation. However, the coverage scores (0.44‐0.45) suggest that while these solutions account for a significant proportion of active initiatives, they do not capture all possible pathways. This highlights that some initiatives remain active through alternative, unexplored mechanisms.

The presence of solutions where ownership models are weak, but governance structures are present, suggests that effective governance can compensate for less formal ownership arrangements. Similarly, while some solutions incorporate financial incentives and sustainability models, others indicate that initiatives can persist without these elements, provided they integrate strong governance or transactional mechanisms. These findings emphasize the diversity of structural configurations that contribute to long-term viability.

#### Implications

The implications of the analysis are as follows:

- Initiatives with weaker ownership structures can persist if alternative governance models are in place.
- Financial incentives are not universally necessary, but their presence in certain configurations suggests they contribute positively.
- Sustainability strategies are not always essential, but those initiatives without them tend to rely on stronger governance or transaction mechanisms.

### Sensitivity Analysis

#### Overview

A sensitivity analysis was conducted by systematically varying the inclusion threshold from 0.60 to 0.80 to assess the robustness of the identified configurations. The results demonstrate how different thresholds impact the number and structure of minimized solutions. The results of this analysis are shown in Table 5.

**Table 5.** Sensitivity analysis.

Inclusion threshold	Minimized configurations	Inclusion score (inclS)	Proportional reduction in inconsistency (PRI)	Solution coverage (covS)
0.6	¬O * ¬B * ¬I * ¬T + ¬O * ¬G * ¬B * ¬T * ¬S + O * ¬G * B * ¬I * ¬S + O * ¬B * ¬I * T * S + G * ¬B * ¬I * ¬T * ¬S + ¬O * G * ¬B * ¬I * S	0.734	0.734	0.577
0.65	¬O * ¬B * ¬I * ¬T + ¬O * ¬G * ¬B * ¬T * ¬S + O * ¬G * B * ¬I * ¬S + O * ¬B * ¬I * T * S + G * ¬B * ¬I * ¬T * ¬S + G * ¬B * ¬I * T * S	0.774	0.774	0.44
0.7	¬O * ¬G * ¬B * ¬I * ¬T + ¬O * ¬G * ¬B * ¬T * ¬S + O * ¬B * ¬I * T * S + G * ¬B * ¬I * ¬T * ¬S + G * ¬B * ¬I * T * S + O * ¬G * B * ¬I * T * ¬S	0.808	0.808	0.395
0.75	¬O * ¬G * ¬B * ¬I * ¬T + ¬O * ¬G * ¬B * ¬T * ¬S + ¬O * ¬B * ¬I * ¬T * ¬S+O * ¬G * ¬B * ¬I * T * S + O * ¬G * B * ¬I * T * ¬S	0.84	0.84	0.243
0.8	¬O * ¬G * ¬B * ¬T * ¬S + O * ¬G * B * ¬I * T * ¬S	0.925	0.925	0.11

#### Summary of Sensitivity Results

The sensitivity results of the analysis are as follows:

- Lower thresholds (0.60‐0.65) produced a greater number of solutions, capturing a broader range of configurations that may contribute to sustained initiative activity.
- At 0.70, some pathways observed at lower thresholds were eliminated, leading to a more refined set of explanatory configurations.
- Higher thresholds (0.75‐0.80) significantly reduced the number of valid pathways, indicating that only the most consistently sufficient conditions remained.

The analysis across multiple inclusion thresholds identified stable configurations that appeared consistently, indicating relationships between governance, ownership, and incentive structures in determining sustained initiative activity. These configurations remained present even as the consistency threshold increased.

As the inclusion threshold increased, the number of configurations decreased, reflecting a more restrictive selection process that excluded cases with lower consistency scores. At a threshold of 0.75, fewer configurations met the criteria, and by 0.80, only two configurations remained. This indicates that while multiple pathways lead to sustained initiative status, only a subset consistently exhibits the highest levels of consistency.

The analysis also showed that the absence of a defined business model (¬B) and financial incentives (¬I) appeared in multiple solutions across different thresholds. This suggests that while financial incentives and structured business models may contribute to sustainability, other mechanisms, such as governance structures and transaction models, can support sustained activity in their absence.

The role of sustainability planning (S) varied across thresholds, indicating that while sustainability mechanisms contribute to long-term viability, they are not always necessary. Some initiatives maintained sustained activity without explicit sustainability planning, suggesting that alternative structural factors, such as governance and transaction mechanisms, may provide sufficient support.

### Interpretation of Findings

The analysis identified multiple configurations of conditions associated with sustained initiative activity. The base model, run at an inclusion threshold of 0.65, produced two primary solutions, each representing distinct pathways leading to continued operation. The presence of multiple valid configurations supports the principle of equifinality, indicating that different combinations of governance, ownership, business, incentive, and transaction models can contribute to sustained activity. The solutions demonstrated that initiatives without a strong ownership structure could persist if supported by governance mechanisms, suggesting that centralized control is not always necessary when alternative oversight structures are in place. Similarly, the absence of a defined business model in several configurations indicates that some initiatives operate successfully without a clear commercial framework, relying instead on cooperative models, external funding, or nonmonetary incentives.

The analysis also highlighted the role of financial incentives and sustainability planning in initiative survival. Some pathways included sustainability mechanisms and incentive structures, reinforcing the idea that structured support mechanisms contribute to long-term viability. However, other solutions demonstrated that initiatives without explicit financial incentives or sustainability frameworks could still maintain activity, particularly if supported by effective governance and transaction models. This suggests that while financial and sustainability considerations may enhance stability, they are not universally necessary for success.

The sensitivity analysis, conducted across multiple inclusion thresholds, provided further insight into the robustness of these findings. At lower thresholds, a broader range of configurations was identified, capturing a wider set of conditions leading to sustained initiative status. As the threshold increased, the number of valid solutions decreased, with only the most consistent configurations remaining at the highest inclusion levels. This pattern indicates that while multiple pathways can lead to continued activity, only a subset demonstrates the strongest and most consistent relationships between conditions and outcomes.

At higher thresholds, the solutions became more restrictive, eliminating cases with lower consistency scores. By the highest inclusion level of 0.80, only two configurations remained, representing the most reliable pathways associated with sustained initiative activity. This suggests that while various factors contribute to initiative sustainability, only a few consistently demonstrate strong explanatory power. The reduction in configurations at higher thresholds reinforces the importance of governance structures and transaction mechanisms as key determinants of continued operation, particularly when financial incentives and sustainability planning are absent.

## Discussion

### Methodology

The methodology used in this study followed a structured multistage approach. First, a snowball sampling technique was used to identify relevant medical data sharing initiatives, with a particular focus on governance, ownership, transaction models, and sustainability. This approach allowed for the inclusion of initiatives that may not be widely documented in academic literature but were actively discussed in industry reports, online databases, and open-source repositories. The initial set of initiatives was identified through keyword searches and industry reports, and additional initiatives were identified through references, expert consultations, and monitoring relevant discussions.

Following identification, each initiative was systematically analyzed across predefined factors, including governance structures, ownership models, business and transaction mechanisms, incentive structures, and sustainability approaches. These attributes were extracted from publicly available documentation, technical white papers, organizational policies, and, where possible, direct engagement with initiative representatives. The initiatives were then categorized using multivalue attributes to facilitate structured comparative analysis.

To assess the operational status of the initiatives, a follow-up analysis was conducted between December 2023 and March 2024. This verification process included reviewing official websites, press releases, regulatory filings, industry reports, and online community discussions. Initiatives were classified as either active or inactive, depending on the presence of ongoing operations, product updates, or sustained engagement.

Finally, fsQCA was applied to identify configurational relationships between the different factors and sustained initiative activity. This approach allowed for an analysis of multiple pathways leading to continued operation rather than assuming a single linear relationship. By systematically comparing different configurations of governance, ownership, incentives, and transaction mechanisms, the analysis identified the strongest structural factors associated with long-term initiative viability.

While the use of snowball sampling can introduce certain biases, it was particularly useful in this context due to the dynamic and rapidly evolving nature of the field of medical data sharing. Many initiatives in this space are emerging and may not be widely documented in traditional academic databases or directories. By consulting a range of sources and engaging with experts and stakeholders in the field, we aimed to capture a comprehensive and nuanced understanding of the current landscape of medical data sharing initiatives. This approach ensured that the review included as many relevant initiatives as possible, even those less visible or established, providing a thorough and representative survey of the field.

The use of fsQCA provided a structured approach to examining the relationships between organizational factors and initiative sustainability. In contrast to variable-based statistical methods, fsQCA identifies multiple configurations leading to an outcome, allowing for the identification of equifinality, where different sets of conditions can result in similar outcomes. This approach was suited to the diverse characteristics of the initiatives studied, which varied across governance structures, business models, and sustainability mechanisms. The calibration of categorical variables into fuzzy-set membership scores allowed for degrees of presence or absence rather than strict binary classifications. A sensitivity analysis across multiple inclusion thresholds was conducted to assess the consistency of the results. This allowed for a structured analysis of relationships between factors while maintaining a focus on individual initiatives.

### Classification of Initiatives

The classification of initiatives by governance, ownership, business, transaction, incentive, sustainability models, and blockchain usage provided a structured basis for analyzing patterns within medical data sharing initiatives. Governance models varied from centralized board-based structures to decentralized user-regulated approaches, reflecting differing levels of oversight and participant control. Ownership structures showed a predominance of private and cooperative models, with some initiatives operating as public-private partnerships or under government control. Business models highlighted distinctions between for-profit, nonprofit, and cooperative models, emphasizing different economic strategies for sustainability. Transaction models ranged from direct payments and tokenized systems to reward-based mechanisms and freely accessible services, demonstrating diverse approaches to financial interactions. Incentive structures incorporated self-interest, financial compensation, and altruistic motivations, illustrating how initiatives attract participation. Sustainability models varied widely, including membership fees, data sales, investment funding, and subscription-based services, indicating different strategies for long-term viability. Blockchain usage was primarily concentrated in initiatives aiming to enhance security, transparency, and data ownership, though some projects operated without blockchain-based frameworks. These classifications allowed for a comparative analysis of how different structural and economic factors contribute to the continued operation and impact of medical data sharing initiatives.

### Analysis of Initiatives

The results of the analysis highlight several key factors associated with the continued activity of medical data sharing initiatives. Governance structures and ownership models emerged as significant influences, with initiatives that incorporated structured governance mechanisms—such as cooperative models, regulatory compliance, or board oversight—more frequently sustaining operations over time. While strong ownership models were not universally necessary, the presence of governance frameworks appeared to compensate for weaker ownership structures. This suggests that clearly defined decision-making and accountability mechanisms contribute to long-term viability, even in the absence of centralized control.

Financial sustainability and incentive structures also played a role in distinguishing active initiatives. The findings indicate that a well-defined business model is not always essential, as some initiatives without clear revenue-generating mechanisms remained operational. However, those that did sustain themselves often incorporated diverse funding strategies, including membership fees, data access sales, or external investment. Incentive models varied widely, with some initiatives relying on direct financial compensation for participation, while others leveraged nonfinancial motivations such as improved health care access or research contributions. The variability in sustainability and incentive strategies suggests that no single approach guarantees success, but initiatives that align their financial model with stakeholder expectations and engagement mechanisms are more likely to persist. Finally, the analysis demonstrated that transaction models, particularly those leveraging token economies or alternative financial structures, were not necessarily a determinant of success but functioned as supporting mechanisms that could enhance participation and sustainability when combined with effective governance and incentive models.

### Limitations

Several limitations should be considered when interpreting these findings. The study analyzed a selective set of initiatives identified through snowball sampling and available documentation. While this approach allowed for a broad survey of relevant initiatives, it may have introduced selection biases, as less visible or undocumented initiatives could have been excluded. The reliance on publicly available information also meant that classification was sometimes based on inference rather than direct confirmation, particularly for governance structures and financial sustainability models. This introduces potential uncertainty, as internal decision-making processes and strategic priorities may not have been fully captured.

Additionally, the rapidly evolving nature of the space means that some findings may not remain applicable over time. New governance models, technological advancements, and shifts in regulatory frameworks could alter the viability of different configurations. Methodologically, QCA is sensitive to case selection and threshold settings, meaning that small changes in the dataset or inclusion criteria could influence the resulting configurations. While sensitivity analysis helped assess the robustness of the findings, the results should be understood as identifying patterns rather than deterministic causal relationships. Expanding the dataset to include a wider range of initiatives, including those without blockchain integration, could provide a more comprehensive perspective on the factors influencing long-term sustainability.

### Future Research

Future research could build on this study by conducting longitudinal analyses to track how governance and business models evolve over time and how they impact long-term viability. Additional qualitative research could provide deeper insights into decision-making processes within initiatives, particularly regarding governance and financial sustainability. Interviews with key stakeholders could help validate the findings and offer further context on the mechanisms driving success or failure.

Further studies could also explore the role of emerging technologies, such as AI-driven data management and decentralized identity frameworks, in shaping the future of medical data sharing. Investigating how different regulatory environments influence the adoption and sustainability of these initiatives would also be valuable. Finally, expanding the dataset to include a broader range of initiatives, particularly those outside blockchain-based models, could provide a more comprehensive understanding of the factors influencing success in the medical data sharing ecosystem.

### Conclusions

Our findings highlight the diverse governance, ownership, and financial sustainability models adopted by medical data sharing initiatives. The results suggest that no single factor determines long-term viability, but rather that different configurations of governance structures, business models, and incentives can contribute to sustained activity. Initiatives with centralized governance structures and clearly defined financial sustainability strategies were more likely to remain active. However, the presence of decentralized governance models and alternative funding mechanisms also demonstrated viability, particularly in cooperative and blockchain-based initiatives. The findings reinforce the importance of aligning governance structures with financial models to ensure long-term sustainability while maintaining transparency and user trust.

The analysis also suggests that financial incentives and transaction mechanisms play varying roles depending on the broader structural configuration of an initiative. While some successful initiatives relied on direct financial incentives, others sustained engagement through cooperative governance, shared decision-making, or intrinsic motivations such as access to health insights. This variability underscores the need for initiatives to tailor their incentive structures to their specific context, balancing financial sustainability with participant engagement and ethical data sharing practices.

## Abbreviations

CDIP: Civic Data Identity Partnership
fsQCA: fuzzy-set qualitative comparative analysis
GDPR: General Data Protection Regulation
mvQCA: multivalue qualitative comparative analysis
QCA: qualitative comparative analysis
SaaS: Software-as-a-Service
